# Spatial Profiles of Phosphate in Roots Indicate Developmental Control of Uptake, Recycling, and Sequestration^1^^[CC-BY]^

**DOI:** 10.1104/pp.20.01008

**Published:** 2020-09-30

**Authors:** Abira Sahu, Swayoma Banerjee, Aditi Subramani Raju, Tzyy-Jen Chiou, L. Rene Garcia, Wayne K. Versaw

**Affiliations:** a Department of Biology, Texas A&M University, College Station, Texas 77843; b Agricultural Biotechnology Research Center, Academia Sinica, Taipei 11529, Taiwan

## Abstract

The availability of inorganic phosphate (Pi) limits plant growth and crop productivity on much of the world’s arable land. To better understand how plants cope with deficient and variable supplies of this essential nutrient, we used Pi imaging to spatially resolve and quantify cytosolic Pi concentrations and the respective contributions of Pi uptake, metabolic recycling, and vacuolar sequestration to cytosolic Pi homeostasis in Arabidopsis (*Arabidopsis thaliana*) roots. Microinjection coupled with confocal microscopy was used to calibrate a FRET-based Pi sensor to determine absolute, rather than relative, Pi concentrations in live plants. High-resolution mapping of cytosolic Pi concentrations in different cells, tissues, and developmental zones of the root revealed that cytosolic concentrations varied between developmental zones, with highest levels in the transition zone, whereas concentrations were equivalent in epidermis, cortex, and endodermis within each zone. Pi concentrations in all zones were reduced, at different rates, by Pi starvation, but the developmental pattern of Pi concentration persisted. Pi uptake, metabolic recycling, and vacuolar sequestration were distinguished in each zone by using cyanide to block Pi assimilation in wild-type plants and a vacuolar Pi transport mutant, and then measuring the subsequent change in cytosolic Pi concentration over time. Each of these processes exhibited distinct spatial profiles in the root, but only vacuolar Pi sequestration corresponded with steady-state cytosolic Pi concentrations. These results highlight the complexity of Pi dynamics in live plants and revealed developmental control of root Pi homeostasis, which has potential implications for plant sensing and signaling of Pi.

## Abstract (precis)

Distinct spatiotemporal patterns for the uptake, recycling, and vacuolar sequestration of phosphate in the root indicate developmental control of cytosolic phosphate homeostasis.

Phosphorus is an essential nutrient that plants acquire, assimilate, and distribute in the form of inorganic phosphate (Pi). Although substantial amounts of Pi are needed for optimal plant growth and productivity, much of the Pi in soil is unavailable because it is immobilized as sparingly soluble complexes or exists in organic forms that are not directly accessible to plants (Schachtman et al., 1998). To cope with low Pi conditions, plants activate a complex array of adaptive responses to enhance Pi acquisition and to recycle and reallocate internal stores. These Pi starvation responses (PSRs) include growth of lateral roots and root hairs to increase the range of and capacity for nutrient scavenging (Bates and Lynch, 1996; Kellermeier et al., 2014), which are extended in many species through the formation of symbiotic association with mycorrhizal fungi (Javot et al., 2007); secretion of organic acids and phosphatases to increase the concentration of available Pi in soil solution (Vance et al., 2003); increased production of Pi transporters at the root-soil interface (Nussaume et al., 2011); modification of metabolic pathways to conserve cellular Pi (Plaxton and Tran, 2011); and redistribution of Pi between organs and subcellular compartments (Mimura, 1999; Raghothama, 1999; Versaw and Garcia, 2017). Signaling mechanisms governing PSRs include responses to external Pi concentrations to modify the root system architecture (López-Bucio et al., 2002; Svistoonoff et al., 2007; Ticconi et al., 2009; Müller et al., 2015; Balzergue et al., 2017) and responses to internal Pi concentrations to maintain Pi homeostasis (Rubio et al., 2001; Thibaud et al., 2010; Puga et al., 2014; Wang et al., 2014; Sun et al., 2016; Chien et al., 2018). How these signaling mechanisms are coordinated is unclear, but Pi itself is regarded as a primary signal (Ticconi et al., 2001; Puga et al., 2014; Jost et al., 2015; Wild et al., 2016). For example, Pi in the cytosol can readily diffuse to the nucleus (Naim et al., 2007) where it or Pi-containing metabolites such as diphosphoinositol phosphates (PP-InsPs) promote interaction between SYG1/Pho81/XPR (SPX)-domain-containing proteins and PHOSPHATE STARVATION RESPONSE (PHR) transcription factors to modulate the activation of many PSR genes (Puga et al., 2014; Wang et al., 2014; Wild et al., 2016; Kuo et al., 2018; Zhu et al., 2019). Together, these observations suggest that the concentration of Pi in the cytosol elicits appropriate responses to changes in Pi availability.

Because the cytosol is the nexus for both intracellular and intercellular Pi distribution, multiple processes must be coordinated to maintain cytosolic Pi concentrations within a relatively narrow, but dynamic range. Initially, Pi uptake is mediated under both Pi-replete and Pi-deficient conditions by members of the PHOSPHATE TRANSPORTER1 (PHT1) family of Pi transporters located in the plasma membrane of root epidermal and cortical cells as well as the root cap (Ayadi et al., 2015; Kanno et al., 2016a). Mutants with defects in PHT1 transporters or their trafficking to the plasma membrane exhibit reduced Pi uptake and diminished overall plant growth and development (Shin et al., 2004; González et al., 2005; Bayle et al., 2011; Ayadi et al., 2015; Kanno et al., 2016a). An innovative ^33^P imaging technique demonstrated that PHT1 transporters in the root cap contribute a substantial portion of total root Pi acquisition (Kanno et al., 2016a). However, the impact of this uptake activity on cytosolic Pi concentrations in the root cap and other parts of the root is unknown because this imaging approach does not distinguish between subcellular locations or between inorganic and organic forms of Pi.

Pi acquired by the root is rapidly assimilated to form ATP via oxidative and substrate-level phosphorylation (Arisz et al., 2009) and then incorporated into macromolecules and metabolites from which Pi is subsequently recycled with varied rates and magnitudes (Plaxton and Tran, 2011). Much of the assimilation and metabolic recycling of Pi occurs in organelles, and intracellular Pi transporters are therefore required to direct Pi between the cytosol and each cell compartment (Versaw and Garcia, 2017). Notably, Pi transport into the vacuole, the largest reservoir of Pi in the cell, is catalyzed by members of the PHOSPHATE TRANSPORTER5 (PHT5) family, which is also called VACUOLAR PHOSPHATE TRANSPORTER (VPT; Liu et al., 2015, 2016; Xu et al., 2019), whereas Pi export from the vacuolar lumen is mediated by members of the VACUOLAR PHOSPHATE EFFLUX (VPE) transporter family (Xu et al., 2019). Mutants with defects in these transporters exhibit altered patterns of vacuolar Pi accumulation consistent with their roles in either import or export, as well as altered expression of PSR genes and reduced overall plant growth, suggesting that these defects also affect cytosolic Pi concentrations (Liu et al., 2015, 2016; Xu et al., 2019). Although the vacuolar Pi pool can help buffer the cytosol against concentration changes (Mimura, 1999; Pratt et al., 2009), this buffering can be exhausted or outpaced by chronic or acute Pi deprivation, respectively, resulting in altered cytosolic Pi concentrations (Rebeille et al., 1984; Pratt et al., 2009; Mukherjee et al., 2015).

Pi is mobile in plants and its distribution between cells in the root, as well as its movement to shoots via xylem and redistribution back to roots via phloem, could also influence cytosolic Pi concentrations within individual root cells. Mechanisms of intercellular movement of Pi within the root vary with development. Cell-to-cell diffusion through plasmodesmata (symplastic transport) is possible in apical parts of the root spanning from the meristematic zone (MZ) to the first root hair-containing region of the differentiation zone (DZ; Duckett et al., 1994; Benitez-Alfonso et al., 2013). However, symplastic connectivity is blocked in the DZ and mature root zone (MR), including root hairs, necessitating transmembrane transport processes to cross the plasma membrane. The transfer of Pi from root to shoot requires export of cytosolic Pi to the xylem apoplastic space, which is mediated by PHOSPHATE1 (PHO1) transporters in cooperation with a subset of PHT1 transporters (Poirier et al., 1991; Hamburger et al., 2002; Stefanovic et al., 2007; Lapis-Gaza et al., 2014). Mutations of *PHO1* cause reduced Pi content in shoots, stunted growth, and strong expression of PSR genes (Poirier et al., 1991; Hamburger et al., 2002). It is presently unclear which transporters are responsible for the redistribution of Pi from shoot to root, but transcript localization data suggest that members of the PHT1 family may be involved (Lapis-Gaza et al., 2014).

The relative contributions of the different processes that influence cytosolic Pi homeostasis and any spatiotemporal variations in these processes within roots are largely unknown. This is primarily due to the difficulty of distinguishing cytosolic Pi from other cellular pools in live plants (Kanno et al., 2016b). We previously reported that ratiometric imaging of a genetically encoded FRET-based Pi sensor stably expressed in Arabidopsis (*Arabidopsis thaliana*) could be used to monitor relative changes in cytosolic Pi concentrations in epidermal cells within the DZ or MR of the root (Mukherjee et al., 2015; Banerjee et al., 2016). Here, we have extended and calibrated this live imaging approach to systematically evaluate absolute, rather than relative, cytosolic Pi concentrations in different tissues and developmental zones of the root under Pi-replete and Pi-deficient growth conditions. We show that cytosolic Pi concentrations differ between developmental zones, with highest levels in the transition zone (TZ), and that this distribution pattern is independent of Pi supply. We also distinguish the contributions of Pi uptake and metabolic recycling to cytosolic Pi levels by blocking Pi assimilation with cyanide. Similar experiments conducted with a vacuolar Pi uptake mutant, *phosphate transporter5;1-2* (*pht5;1-2*; Liu et al., 2016), suggest that developmental differences in vacuolar sequestration are responsible for the observed pattern of Pi distribution in the root.

## RESULTS

### Spatial Analysis of Cytosolic Pi Concentrations in the Root Under Pi-replete Growth Conditions

**Figure 1.**
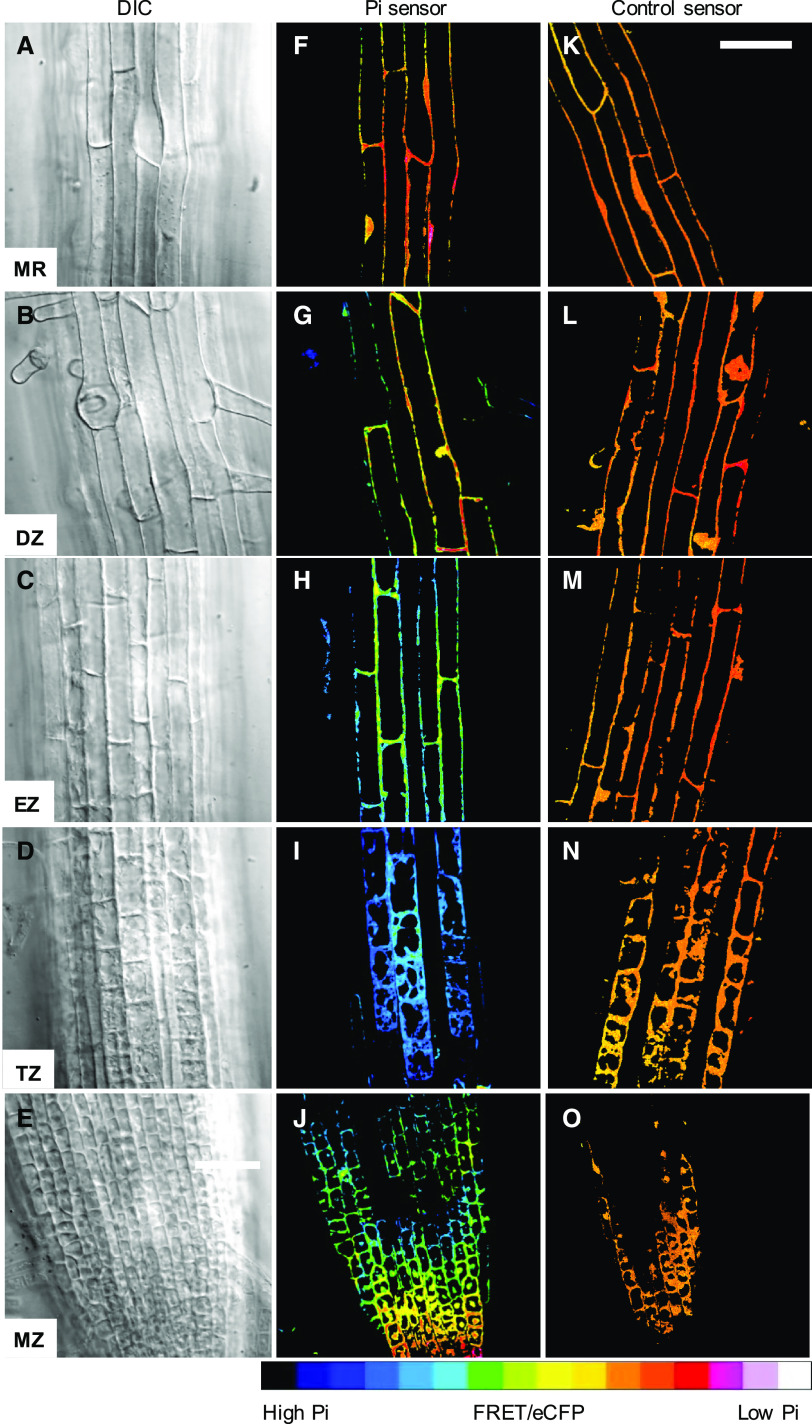
Ratiometric imaging of cytosolic Pi levels in Arabidopsis roots. Shown are differential interference contrast (DIC; A–E) and emission ratio micrographs (F–O) of five developmental zones, the MZ, TZ, EZ, DZ, and MR. Emission ratio images are representative of roots with a Pi sensor (F–J) and a control sensor that is unresponsive to Pi (K–O). Scale bars = 50 μm.

We hypothesized that cytosolic Pi concentration is not uniform in the root due to spatial differences in Pi uptake (Kanno et al., 2016a) and metabolic demands for Pi, e.g. cell division in the root apical meristem (Lai et al., 2007). To explore this possibility, we first examined relative cytosolic Pi levels in epidermal cells throughout the developing and mature roots of seedlings grown under Pi-replete (0.25 mm) conditions. Seedlings that constitutively express a cytosol-localized, FRET-based Pi sensor (cpFLIPPi-5.3m; Mukherjee et al., 2015) were grown for 6 d, which surpassed the time after germination needed to establish root meristem size (Di Mambro et al., 2017). We then used ratiometric imaging (emission ratio of the circularly permutated Venus/enhanced cyan fluorescent protein [cpVenus/eCFP] pair) as described previously (Banerjee et al., 2016) to assess relative Pi concentrations in each developmental zone of the root, namely the MZ, the TZ, the elongation zone (EZ), the DZ, and the MR (Baluška et al., 2001; Verbelen et al., 2006; Petricka et al., 2012). Representative images (Fig. 1) and the corresponding data (Supplemental Fig. S1) revealed that Pi-dependent emission ratios varied between root zones with a consistent pattern in which cells in the TZ had the highest relative Pi levels. We attributed differences in emission ratio to Pi concentrations rather than nonspecific effects of cellular environments or to imaging artifacts, because no spatial variation in emission ratio was detected for a Pi-insensitive control sensor (cpFLIPPi-Null; Fig. 1; Supplemental Fig. S1; Banerjee et al., 2016). Similarly, sensor protein abundance, as indicated by fluorescence emission, was comparable throughout the root (Supplemental Fig. S1) and was also equivalent to previous independent transformants (Banerjee et al., 2016). Although these results supported our initial hypothesis of nonuniform Pi distribution in the root, the developmental pattern was surprising, because cells in the MZ, TZ, and EZ are symplastically connected (Duckett et al., 1994; Benitez-Alfonso et al., 2013).

To determine absolute, rather than relative, cytosolic Pi concentrations, we needed to calibrate Pi sensor emission ratios. In situ calibration has been used for other FRET-based ion sensors (Shen et al., 2013; Carter et al., 2017), but we were unable to use this approach, because no suitable ionophore and membrane-permeable chelator for Pi was available. We found that in vitro calibration was also unsuitable, because emission ratios measured in roots were consistently outside the saturation limit measured in vitro despite the use of identical imaging conditions and regardless of assay buffer composition (Mukherjee et al., 2015), suggesting that the cell wall or other cell materials may unequally influence, e.g. quench, emission from the two fluorescent protein components of the sensor. We therefore employed a microinjection strategy to directly manipulate cytosolic Pi concentration and then measure emission ratio within the injected cell.

**Figure 2.**
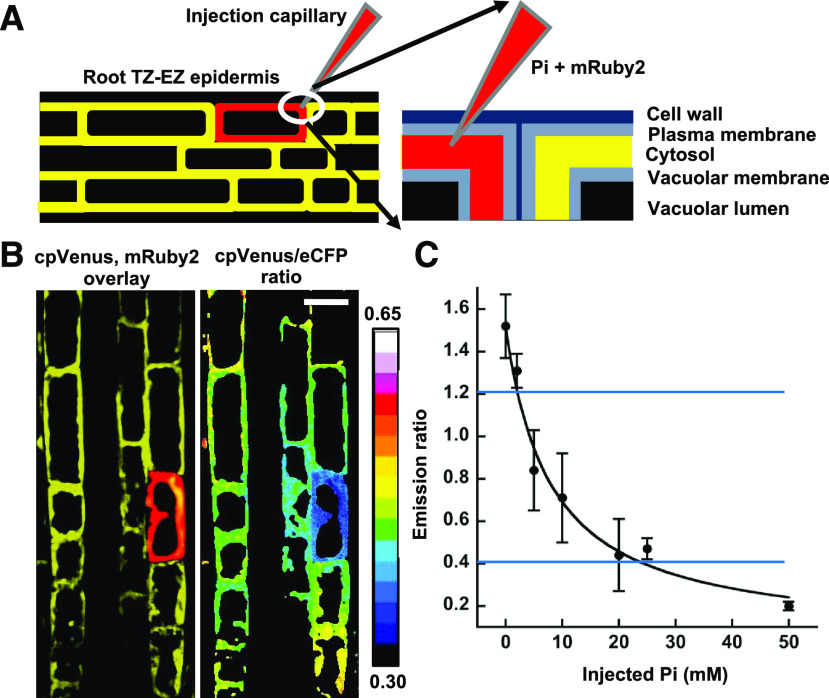
In vivo calibration of Pi sensor emission ratios. A, Schematic representation of microinjection of a root epidermal cell in the TZ located near the EZ junction. Red indicates the mRuby2 marker, which demarcates the injected cell. The inset shows the capillary tip positioned in the cytosol. B, Fluorescent micrographs of cpVenus emission merged with mRuby2 emission (left) and the Pi sensor emission ratio (cpVenus/eCFP) after injection. The color scale represents emission ratio values. Scale bar = 20 μm. C, In vivo calibration curve for Pi sensor emission ratios. Plotted values are mean ± sd emission ratios obtained from six to eight individual injections. Blue lines indicate limits for emission ratio values observed in uninjected transgenic plants.

Epidermal cells located in the TZ were injected with solutions buffered at cytosolic pH (7.3; Shen et al., 2013) that contained defined Pi concentrations, potassium gluconate as needed to maintain constant potassium ion concentration, and the fluorescent protein mRuby2 as a marker, which had no measurable effect on Pi sensor emission ratios. To ensure that cytosolic Pi concentrations closely matched injected concentrations, including those below endogenous levels, we injected volumes 16- to 20-fold greater than the average cytosolic volume of the target cells (Fig. 2A; Table 1). As shown in Figure 2B, the mRuby2 marker remained confined within the cytosol of the injected cell; however, altered emission ratios in adjacent cells suggested that Pi in the excess injection buffer flowed into these cells via plasmodesmata (Duckett et al., 1994; Benitez-Alfonso et al., 2013). Cells were not visibly damaged by injection, and emission ratios returned to the preinjection value within 5 min. To minimize variation in Pi concentrations as the cells reset, we imaged cells within 1.5 s after injection. The resulting data were fit to a single-site binding isotherm to generate an in vivo calibration curve (Fig. 2C) that encompassed all endogenous cytosolic Pi emission ratio values measured in this study. The dissociation constant (*K*_d_) of 7.4 ± 1.7 mm was the same as that measured in vitro (7.4 ± 0.5 mm), suggesting that the sensor was unaffected by the cellular environment. Because this ligand binding assay is nonlinear, its concentration-dependent response is limited at the upper and lower ends of the curve. We therefore restricted estimates of Pi concentrations to those within 20% and 80% saturation, which correspond to 1.6 and 19 mm, respectively. Moreover, if we assume that the 23% relative error for *K*_d_ is uniform within this assay range, then it reflects the accuracy of Pi concentration estimates.

**Table 1.** Volume of cytosol in root epidermal cells Volume is expressed as the mean ± sd of voxel counts in image Z-stacks of 4 to 15 cells in three to five independent plants.

Location	Growth Condition	
	Pi-Replete	Pi-Starved
	*Volume* (*µm*^*3*^)	*Volume* (*µm*^*3*^)
MR	4,976 ± 1,856	2,419 ± 1,074
DZ	4,233 ± 834	2,198 ± 917
EZ	3,660 ± 1,599	1,735 ± 377
TZ	1,213 ± 354	403 ± 131
MZ	473 ± 168	192 ± 97
LRC	810 ± 180	597 ± 98

**Figure 3.**
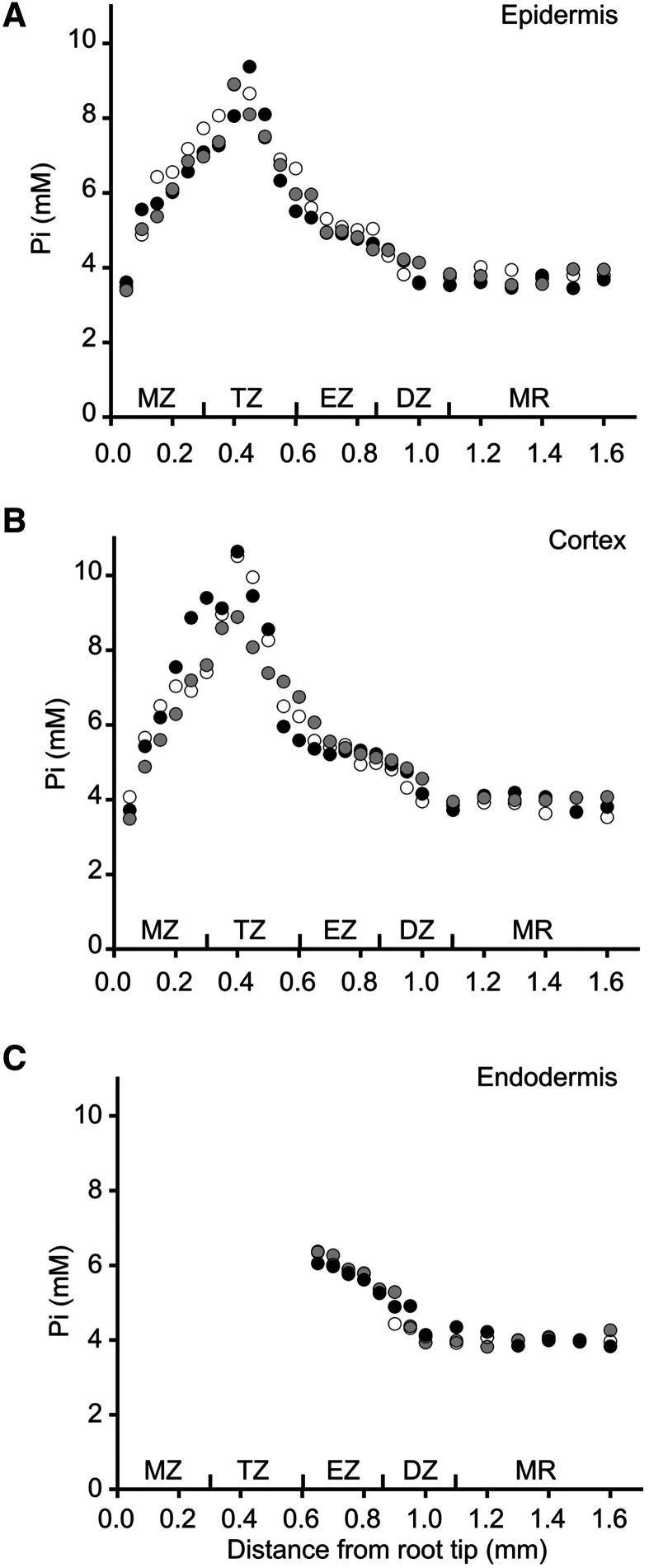
Quantification of cytosolic Pi concentrations in root tissues and developmental zones. Cytosolic Pi concentrations were measured at 50-μm increments from the root tip to the beginning of the MR in epidermal (A), cortical (B), and endodermal (C) cells of three independent plants, represented by white, gray, and black dots. There were no significant differences between individuals or tissues at a given location by ANOVA and Tukey HSD (*P* > 0.05).

We used in vivo-calibrated Pi sensor readout to systematically quantify cytosolic Pi concentrations in different tissues and developmental zones of the root. Optical sections were captured in 50-µm increments from the root tip to the MR at multiple depths to distinguish Pi concentrations in the epidermis, cortex, and endodermis. No substantial differences in emission ratios were detected over the length of the root in plants expressing the control sensor, as in Figure 1, or at any of the tested imaging depths. In contrast, independent plants expressing the Pi sensor exhibited a consistent distribution pattern, with highest cytosolic Pi concentrations in the TZ (∼10 mm) and lowest concentrations in the MZ and MR (3.5 to 4 mm), whereas Pi concentrations were the same in each tissue within a given developmental zone (Fig. 3). However, we were unable to measure cytosolic Pi in the endodermis in the youngest portion of the root because we could not detect fluorescence from the sensor in this region regardless of whether the sensor was expressed from the Arabidopsis *UBQ10* or *CaMV35S* promoters.

**Figure 4.**
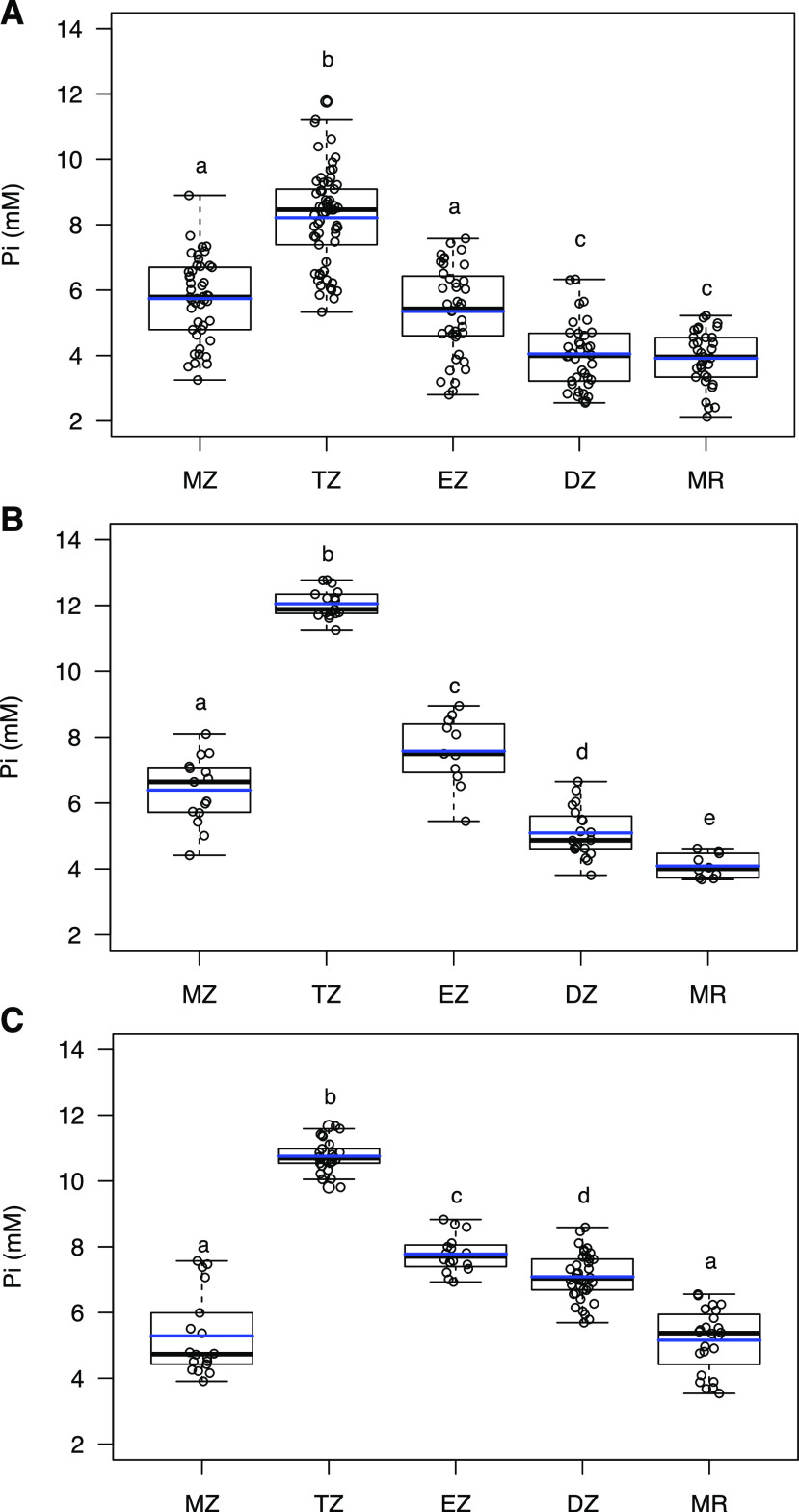
Cytosolic Pi concentrations in roots of seedlings and mature plants. Cytosolic Pi concentrations were quantified in roots of seedlings (A) and primary (B) and lateral (C) roots of mature plants. Arabidopsis plants were grown in Pi-replete medium for 6 d (seedlings) or 3 weeks (mature plants). Pi concentrations were measured in root epidermal cells of five independent plants. Blue and black lines in the box plots denote the mean and median, respectively. Statistically significant differences by ANOVA and Tukey’s HSD (*P* < 0.05) between group means are indicated by lowercase letters.

To determine whether the spatial Pi concentration profile we observed in seedlings was also present in mature plants, we grew plants in agar medium for 3 weeks. At that time, plants had 9 to 10 true leaves and primary root lengths of about 10 cm. We then imaged epidermal cells in each developmental zone in both primary and lateral roots. The Pi distribution pattern in primary and lateral roots (Fig. 4) was similar to that observed in seedlings, suggesting that distribution is developmentally programmed.

### Effect of Pi Availability on Cytosolic Pi Concentrations in the Root

**Figure 5.**
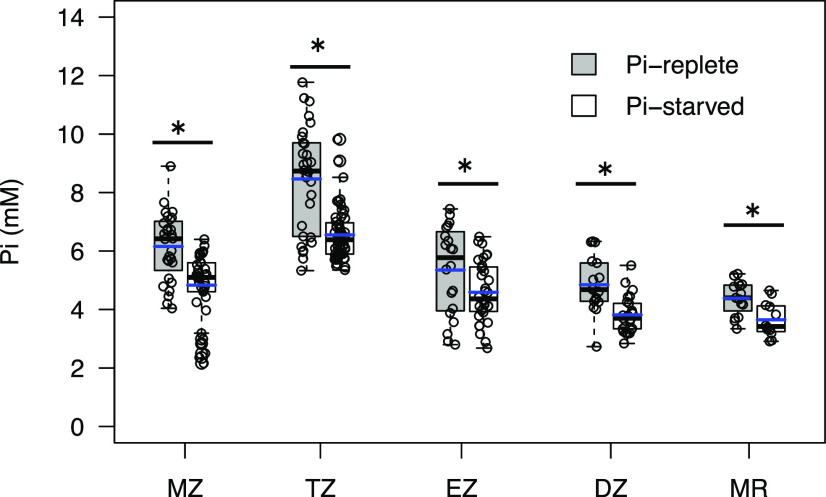
Effect of Pi deprivation on cytosolic Pi concentrations. Seedlings were grown in Pi-replete conditions for 6 d then transferred to the same medium or to medium lacking Pi (Pi-starved) for an additional 2 d. Pi concentrations were measured in epidermal cells of five to seven independent plants (28–37 cells for the MZ, 30–57 cells for the TZ, 20–32 cells for the EZ, 17–25 for the DZ, and 15 cells for the MR). Blue and black lines in the box plots denote the mean and median, respectively. Pi concentrations in all developmental zones of Pi-starved plants are significantly lower than those in plants held in Pi-replete conditions by Student’s *t* test (*P < 0.05).

We observed previously that relative cytosolic Pi levels in the DZ and MR decreased when plants were starved for Pi (Mukherjee et al., 2015; Banerjee et al., 2016). We therefore sought to determine the magnitude of these changes in absolute Pi concentration and to test whether this response also occurs in other parts of the root. Seedlings were grown for 6 d in Pi-replete medium and then transferred to either fresh replete medium or to medium lacking Pi (Pi-starved) for an additional 48 h. Previous experiments demonstrated that this starvation regime is sufficient to induce other PSRs but does not initiate cell death, which is observed when deprivation is prolonged (Mukherjee et al., 2015). Cytosolic Pi concentrations were measured in epidermal cells within each root developmental zone. As shown in Figure 5, Pi starvation reduced cytosolic Pi concentrations in all developmental zones. The reduction was greatest in the TZ (2.6 mm) and least in the DZ (1.1 mm). However, changes in each developmental zone were proportional to the respective initial concentrations, so the distribution pattern with peak concentrations in the TZ was maintained.

**Figure 6.**
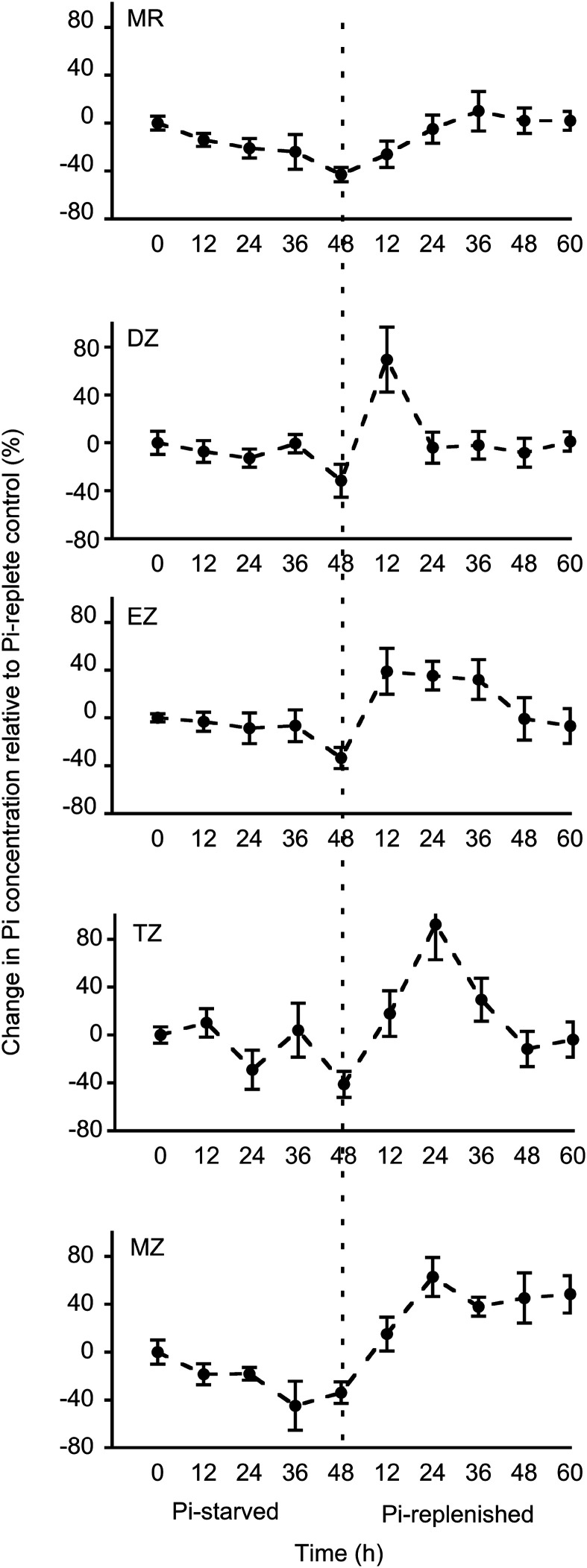
Spatiotemporal dynamics of cytosolic Pi concentrations during changes in Pi supply. Seedlings were grown in Pi-replete medium for 6 d, then transferred to medium lacking Pi (Pi-starved) for 48 h, and then transferred back to Pi-replete medium (Pi-replenished) for 60 h. Epidermal root cells were imaged every 12 h. A control group of plants were maintained in Pi-replete medium. Plotted values are mean ± sd percent changes in Pi concentration relative to control plants, with six to eight independent plants in each group. The dashed vertical line indicates the transition point from Pi-starved to Pi- replenished.

We next asked if there are also temporal differences in responses to Pi starvation and to subsequent replenishment. Seedlings were grown and then starved for Pi for 48 h as described above and then returned to Pi-replete medium for an additional 60 h. Cytosolic Pi concentrations were measured every 12 h and compared to those in plants that were held in Pi-replete medium (Fig. 6). Cells in the MZ and MR were the first to respond to Pi starvation, with a reduction in cytosolic Pi concentration detected in 12 h that continued to steadily decrease throughout the treatment, suggesting that cells in these developmental zones are most sensitive to Pi deprivation. In contrast, Pi levels in the EZ and the DZ showed the highest tolerance to Pi deprivation, with cytosolic Pi levels remaining constant for 36 h and a decline detected only after 48 h. Surprisingly, cytosolic Pi levels in cells within the TZ fluctuated during the starvation treatment, with low concentrations at both 24 h and 48 h.

Although cells in the EZ and DZ appeared to be the most tolerant to Pi deprivation, these cells showed the fastest response to Pi replenishment, with hyperaccumulation of Pi beyond predeprivation levels within 12 h. Pi levels in the EZ declined slowly over the next 48 h, whereas Pi levels in the DZ returned to baseline within an additional 12 h. Hyperaccumulation of cytosolic Pi also occurred in cells within the MZ and TZ, but unlike cells in the EZ and DZ, it took 24 h to reach peak values. High Pi concentrations persisted in the MZ throughout the remainder of the experiment, whereas Pi levels in the TZ returned to baseline within an additional 24 h. Cells in the MR were the slowest to respond to replenishment and reattained baseline concentrations after 24 h. These varied spatiotemporal responses to Pi deprivation and replenishment suggest specialization of mechanisms controlling cytosolic Pi concentration in each developmental zone of the root.

### Spatial Analysis of Pi Recycling and Uptake in the Root

**Figure 7.**
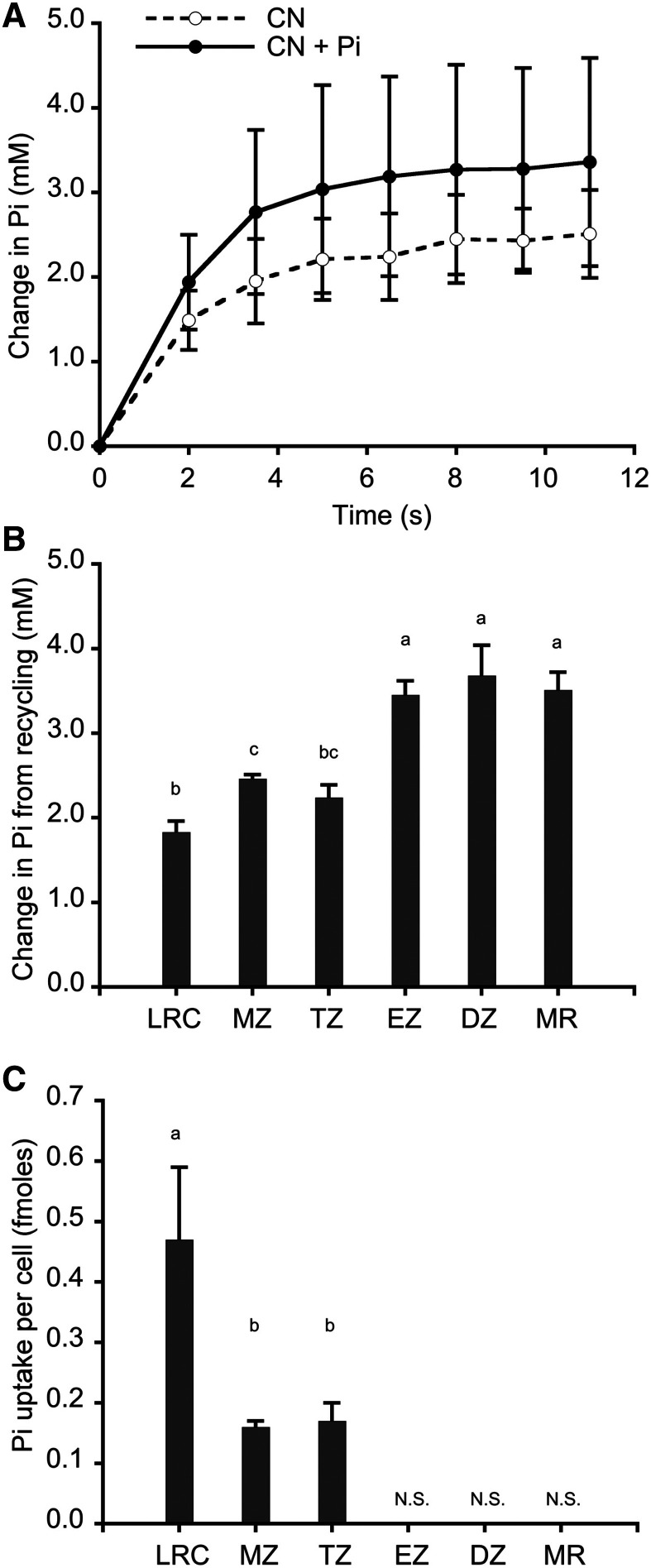
Spatial analysis of Pi recycling and uptake in roots. Seedlings were grown in Pi-replete medium for 6 d then transferred to medium lacking Pi (Pi-starved) for 48 h. A, Change in cytosolic Pi concentrations in epidermal MZ cells when treated with either 10 mm CN or 10 mm CN plus 0.5 mm Pi, which reflect recycling and recycling plus uptake, respectively. Plotted values are mean ± sd changes in Pi concentration at each time point for six independent plants. B, Change in cytosolic Pi concentration from metabolic recycling. Plotted values are mean ± sd changes in Pi concentration after CN treatment in six to eight independent plants (10 cells for the LRC, 8 cells for the MZ, 16 cells for the TZ, 16 cells for the EZ, six cells for the DZ, and six cells for the MR). Statistically significant differences between group means were determined by ANOVA (*P* < 0.05). A Tukey’s HSD test showed that Pi recycling in the TZ is significantly less than in the EZ, DZ, and MR, but not significantly different from in the LRC and MZ. C, Pi uptake calculated from differences in Pi concentrations after CN treatment with and without external Pi, with values corrected for differences in cytosolic volume. Plotted values are mean ± sd Pi uptake (fmoles) per cell during the 10-s assay period. Data were collected from three to seven independent plants. There are statistically significant differences (*P* < 0.05) between group means, as determined by ANOVA and Tukey’s HSD test. Different letters indicate significant differences. No significant uptake (N.S.) was detected in the EZ, DZ, and MR.

Because cellular Pi is rapidly assimilated to ATP and subsequently recycled from ATP and other organic-P molecules at unknown rates (Arisz et al., 2009; Plaxton and Tran, 2011), it has not been possible to resolve the individual contributions of uptake, assimilation, and metabolic recycling to cytosolic Pi concentration. Moreover, the effects of any spatial variations in these processes within the root would be diluted, if not fully masked, in steady-state conditions due to symplastic and vascular transport. However, we reasoned that we could distinguish the initial contributions of Pi uptake and recycling, and also identify potential spatial differences in these processes, through a novel application of our Pi imaging approach. Specifically, we expected that if we used cyanide (CN) to rapidly inhibit the assimilation of Pi to ATP (Van Heyningen, 1935), we would see increased cytosolic Pi levels in the absence of external Pi primarily due to metabolic recycling (hydrolysis of Pi from organic-P molecules). Similarly, if Pi was supplied to a CN-treated root then we predicted that cytosolic Pi concentrations would increase further due to uptake, and we could determine the contribution of this process by difference. To test this strategy, we grew plants with our standard Pi-starvation regime to maximize uptake activities. Preliminary and related experiments indicated that 10 mm CN was sufficient to induce a maximum change in cytosolic Pi concentration (Banerjee et al., 2015) with no nonspecific effects detected with the Pi-insensitive sensor, and that cell viability, as assessed by SYTOX orange (Truernit and Haseloff, 2008; Mukherjee et al., 2015), was unaffected, even when the treatment was continued for 2 min. As shown in Figure 7A, CN treatment in the absence of external Pi induced a rapid increase in cytosolic Pi levels that we attribute to recycling. Pi concentrations consistently reached a maximum within 8 to 10 s, which presumably reflects the time needed to exhaust the Pi recycling capacity of the cell and/or the existing ATP pool. To compare Pi recycling activities in different parts of the root we measured the maximum CN-induced changes in cytosolic Pi concentrations in the lateral root cap (LRC) and in epidermal cells within each developmental zone (Fig. 7B). We found that Pi recycling occurs at a lower level in the LRC, MZ, and TZ and a higher level in the EZ, DZ, and MR. However, these activities did not correspond with the respective cytosolic Pi concentrations (Fig. 5), indicating that recycling is not a major determinant of the developmental pattern of Pi distribution.

To distinguish the contribution of uptake to cytosolic Pi levels, we treated seedlings with CN as described above but also included external Pi (0.5 mm). As shown in Figure 7A, the combination of CN plus Pi resulted in higher cytosolic Pi concentrations than with CN alone, as we predicted, and the difference in Pi concentrations for these two treatments reflects newly acquired Pi. The differences in Pi levels were equivalent regardless of whether CN and Pi were added together or sequentially, indicating that CN had no measurable effect on uptake during the time span of the assay. To compare Pi uptake activities in different parts of the root we measured maximum CN-induced changes in cytosolic Pi concentrations in the presence and absence of external Pi, and then calculated the differences. We detected equivalent changes in Pi concentrations indicative of Pi uptake in the LRC and MZ (0.78 ± 0.07 and 0.84 ± 0.02 mm, respectively), a lower activity in the TZ (0.47 ± 0.07 mm), and no significant changes in the EZ, DZ, and MR. The failure to detect Pi uptake in basal portions of the root is inconsistent with previous findings from radioisotopic Pi uptake experiments (Bates and Lynch, 2000; Kanno et al., 2016a). However, our assay did not account for differences in cytosolic volume. As a result, larger cells with more cytosol would require greater uptake to induce an equivalent change in Pi concentration. Because accounting for cytosolic volumes in cells that exhibited a change in Pi concentration (LRC, MZ, and TZ) would provide a more accurate comparison of uptake activities, we measured cytosolic volumes from voxel counts in image Z-stacks (Table 1), then calculated uptake as the amount of Pi acquired during the 8 s needed to achieve the maximum change in concentration. As shown in Figure 7C, Pi uptake was greatest in the LRC (0.47 ± 0.12 fmoles), and less active in the MZ and TZ (0.16 ± 0.01 and 0.17 ± 0.03 fmoles, respectively). If Pi uptake occurred in the EZ, DZ, and MR at a rate equal to that in the TZ, then the corresponding change in Pi concentration would have been at or below the limit of detection, given the larger cytosolic volumes in these zones. Therefore, we can only conclude that Pi uptake in these regions occurs at a rate that is equal to or less than that in the TZ. Regardless, uptake activities measured in the LRC, MZ, and TZ did not correspond with the respective cytosolic Pi concentrations (Fig. 5), indicating that spatial differences in Pi uptake are not directly responsible for the observed developmental pattern of cytosolic Pi concentrations in the root.

### Effect of Vacuolar Sequestration on Cytosolic Pi Concentrations in the Root

**Figure 8.**
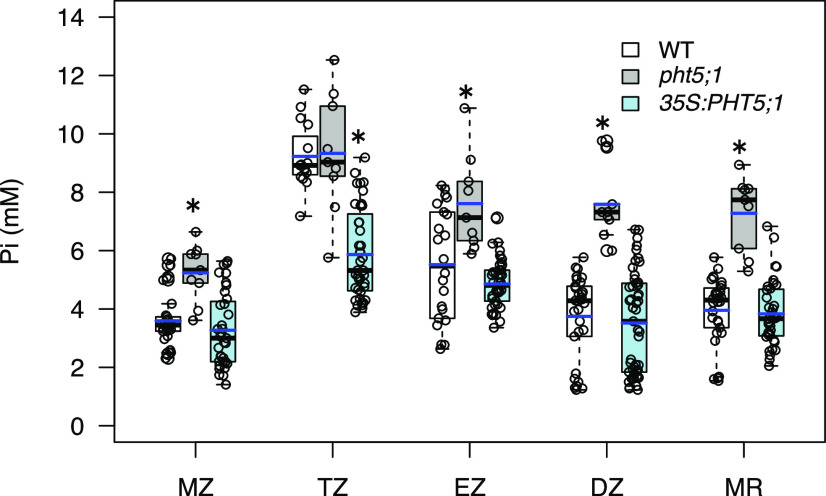
Effect of vacuolar sequestration on cytosolic Pi concentrations in the root. Seedlings were grown in Pi-replete medium for 6 d then imaged. Cytosolic Pi concentrations were significantly greater in *pht5;1-2* plants than in the wild type (WT) in all zones except the TZ (*P < 0.05, Student’s *t* test), whereas in *35S:PHT5;1* plants, cytosolic Pi levels were significantly lower than in the wild type (*P* < 0.05) only in the TZ. Blue and black lines in the box plots denote the mean and median, respectively, for 5 to 16 independent plants (9–35 cells for the MZ, 9–40 cells for the TZ, 9–41 cells for the EZ, 9–49 cells for the DZ, and 9–41 cells for the MR).

Given that vacuoles are the primary storage compartment for Pi in plant cells (Mimura, 1999; Yang et al., 2017), we hypothesized that spatial differences in cytosolic Pi concentrations in the root are the result of developmental differences in its vacuolar sequestration. That is, regions with less vacuolar Pi sequestration would exhibit higher cytosolic Pi concentrations. To test this idea, we compared cytosolic Pi concentrations in each developmental zone of the root in wild-type plants, a vacuolar Pi import-defective mutant, *pht5;1-2*, and a transgenic line in which the PHT5;1 transporter is overexpressed (*35S:PHT5;1*; Liu et al., 2016). Although Arabidopsis has three PHT5 transporters, PHT5;1 has the greatest activity (Liu et al., 2016; Luan et al., 2019). All plants were grown under Pi-replete conditions for 6 d then imaged to determine steady-state cytosolic Pi concentrations. The expected reduced and elevated vacuolar Pi accumulation phenotypes of *pht5;1-2* and *35S-PHT5;1* plants, respectively, were confirmed by evaluating cytosolic Pi concentrations in the MR (largest portion of the root) together with total Pi measured in root extracts (Supplemental Table S1). However, as shown in Figure 8, Pi accumulated in the cytosol to significantly higher concentrations in *pht5;1* than in the wild type in all developmental zones except the TZ. In contrast, Pi concentrations in the TZ were significantly lower in the *35S::PHT5;1* line than in the wild type, but in all other zones, concentrations were similar. These results suggest that in wild-type plants, less vacuolar Pi sequestration occurs in the TZ than in other developmental zones.

**Figure 9.**
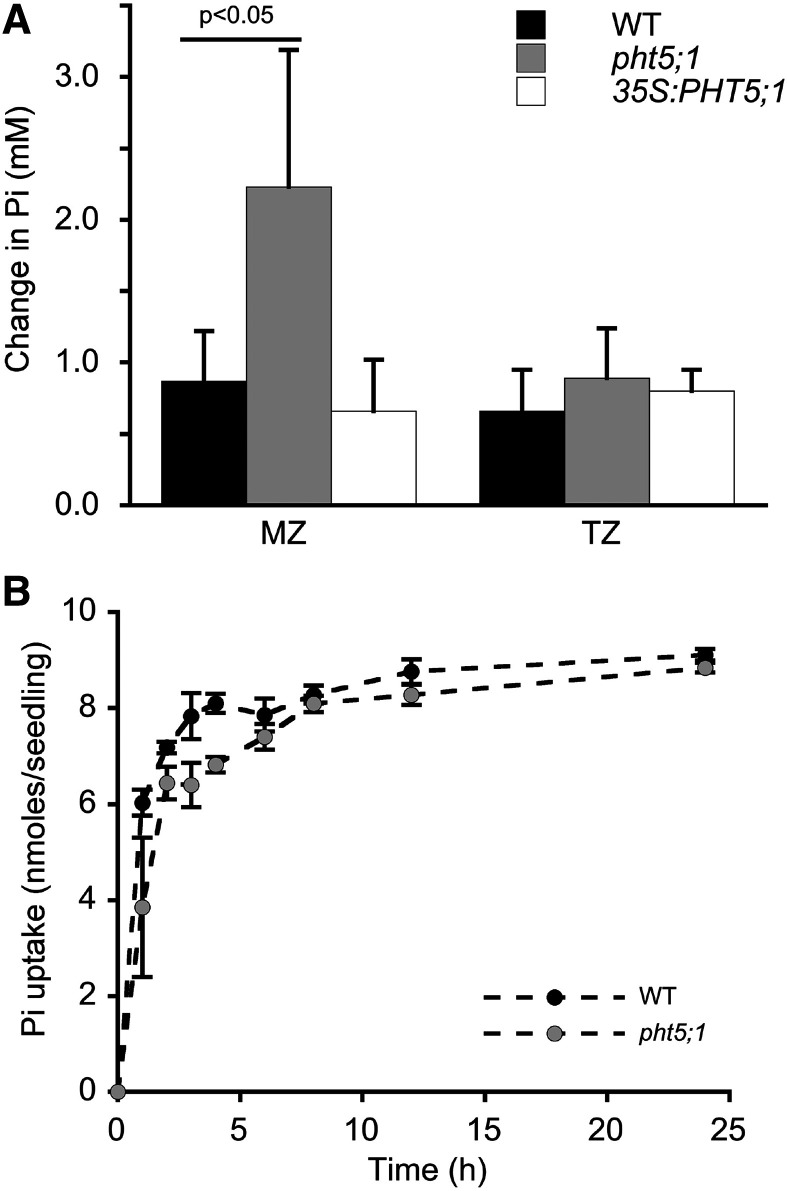
Effects of Pi uptake and vacuolar sequestration on cytosolic Pi concentration. A, Wild-type (WT) and *pht5;1-2* seedlings were grown in Pi-replete medium for 6 d, then transferred to medium without Pi (Pi-starved) for 48 h. Seedlings were sequentially treated with CN and then CN plus Pi, and the changes in cytosolic Pi concentrations after each treatment were measured. Differences in Pi concentrations (mean ± sd for 5–10 independent plants, 8–13 MZ cells, and 9–13 TZ cells) between these treatments reflect the combined activities of Pi uptake and vacuolar Pi sequestration. The difference between the wild type and *pht5;1* in the MZ was significant by Student’s *t* test (*P < 0.05). B, Uptake of external Pi by the entire root. Wild-type and *pht5;1* seedlings were grown for 6 d in Pi-replete medium, then transferred to medium without Pi (Pi-starved) for 48 h. Seedlings of each genotype were then transferred in triplicate groups of 12 to single wells of a 12-well plate containing Pi-replete medium, and over time aliquots were withdrawn for Pi measurement. Pi uptake was calculated from the depletion of Pi in the medium. Plotted values are mean ± sd nanomoles of Pi per seedling from four independent groups of each genotype. There is no significant difference in Pi uptake activities for these genotypes by Student’s *t* test (*P* > 0.05).

To more directly estimate spatial differences in vacuolar Pi sequestration, we focused on cells in the MZ and TZ because these zones showed significant differences in Pi sequestration (Fig. 8) but similar levels of recycling and uptake (Fig. 7, B and C). Wild-type and *pht5;1-2* seedlings were grown with our standard Pi-starvation regime, then treated with CN in the absence of external Pi. We monitored the increase in cytosolic Pi concentration until it reached saturation, which yielded changes in Pi concentration due to recycling as in Figure 7B. We then added CN + 0.5 mm Pi to the same seedlings and again monitored the increase in cytosolic Pi concentration until it reached saturation. The difference in Pi concentrations for these treatments indicated combined activity of uptake and vacuolar sequestration (Fig. 9A). The contribution of vacuolar sequestration could then be estimated by comparing the changes in cytosolic Pi concentration in the wild type to those in the *pht5;1* mutant. For cells in the MZ, changes in Pi concentration due to vacuolar sequestration were nearly equal to those due to uptake, indicating that a substantial fraction of newly acquired Pi is shuttled into vacuoles at a rate that is comparable to that of cellular uptake. This difference was much smaller for cells in the TZ, which is in agreement with results from steady-state measurements (Fig. 8), indicating that significantly less vacuolar Pi sequestration occurs in this zone despite uniform expression of *PHT5;1* throughout the developing root (Brady et al., 2007). We also evaluated vacuolar Pi sequestration in *35S::PHT5;1* plants, but we detected no effect of *PHT5;1* overexpression on cytosolic Pi levels in either the MZ or the TZ (Fig. 9A). It is possible that overexpression of *PHT5;1* augments vacuolar Pi sequestration at a rate that is below the limit of detection for the short-term assay.

It seemed unlikely that increases in cytosolic Pi concentrations in the *pht5;1* mutant that we attributed to reduced vacuolar sequestration (Figs. 8 and 9A) were instead due to greater Pi uptake in this mutant background, because total Pi accumulation was reduced in *pht5;1-2* when plants were grown under replete conditions (Supplemental Table S1), which is consistent with reduced expression of PHT1 transporter genes in this mutant (Liu et al., 2016). However, under Pi-starved conditions, total Pi accumulation in *pht5;1-2* was slightly elevated compared to that in the wild type (Supplemental Table S1). To compare Pi uptake under these conditions more directly, we treated plants with our Pi-starvation regime, as in Figure 9A, then transferred plants to Pi-replete medium and measured the depletion of Pi from the medium as a readout of uptake (Fig. 9B). Pi concentrations in the media decreased at equal rates and magnitudes for the wild type and *pht5;1*, indicating that Pi uptake was not significantly altered by the *pht5;1* mutation under these conditions. Collectively, these results suggest a developmental difference in vacuolar Pi sequestration, with less activity in cells in the TZ resulting in greater cytosolic Pi concentrations in this zone.

## DISCUSSION

The availability of Pi in most soils is suboptimal for crop growth and productivity, and this has led to the widespread use of Pi-containing fertilizers. However, this practice is not economically or environmentally sustainable (Cordell and White, 2014). A comprehensive understanding of how plants acquire and use Pi is therefore needed to optimize agricultural practices and to develop cultivars that are less reliant on Pi fertilizers to obtain high yields. Toward this goal, we developed a dynamic, high-resolution map of cytosolic Pi concentrations in the root, as well as the means to distinguish the contributions of distinct processes that influence cytosolic Pi homeostasis, i.e. uptake, metabolic recycling, and vacuolar sequestration.

Ratiometric imaging of a genetically encoded FRET-based Pi sensor that was constitutively expressed in Arabidopsis revealed that cytosolic Pi concentrations are not uniform in the primary root (Fig. 1). However, as with other sensors that report ratiometric or normalized fluorescence values, this approach yielded relative, rather than absolute, measures of Pi concentration, so we could not define the magnitude of the observed differences. Estimates of Pi concentration based on an in vitro calibration were unreliable because emission ratio values measured in plants were outside the calibration limits. This incongruity was eliminated when we used in vivo calibration, which suggests that the cell wall or other cell components unequally affect, e.g. quench, fluorescence emission from the two fluorescent protein portions of the sensor. Caution is therefore warranted when making estimates of in vivo concentrations of analytes from in vitro calibrations of fluorescence-based sensors.

In vivo calibration of Pi sensor emission ratios relied on microinjection to dilute and displace endogenous cytosolic Pi with buffers containing defined Pi concentrations coupled with rapid imaging to minimize the effects of compensatory processes. We used the resulting calibration curve to estimate endogenous cytosolic Pi levels throughout the root and found that these ranged from a low of 3 mm in the MZ and MR under Pi-starved conditions to a maximum of 12 mm in the TZ under Pi-replete conditions. These concentrations agree with estimates derived from enzyme kinetic studies in which Pi is a substrate or effector (Stitt and Heldt, 1985; Copeland and Zammit, 1994; Volkert et al., 2014), as well as studies using ^31^P NMR spectroscopy with plant cell suspensions (Gout et al., 2011), although values as low as 60 μm have been reported (Pratt et al., 2009). Lower Pi levels can be attained with prolonged Pi starvation, but we observed previously that this also led to substantial cell death under our growth conditions (Mukherjee et al., 2015). Microinjection is technically challenging and requires specialized equipment, which limits the utility of this approach for others. However, because there is little variation in Pi concentration profiles between plants (Fig. 3), we suggest that Pi sensor-expressing plants and associated imaging control plants grown under the same conditions described here could be used as references for other imaging systems. However, the relative magnitudes of emission ratios and overall assay sensitivity can vary substantially with different optics and light detection platforms, so efficacy must be determined empirically. Moreover, it may be necessary to apply confocal techniques to minimize differences in light scattering due to variations in root thickness, maximize the fluorescence signal-to-noise ratio, and define any location-specific spectral corrections (Banerjee et al., 2016).

Although cytosolic Pi concentrations diminished in all developmental zones of the root when plants were starved for Pi, reductions were proportional, so the overall pattern, with highest concentrations in the TZ, was maintained. However, when we examined temporal changes in cytosolic Pi concentrations during Pi deprivation and subsequent resupply, we found that responses differed between zones. For example, cells in the EZ and DZ had the slowest response to Pi starvation, suggesting that they are relatively insensitive to Pi deprivation, but these cells also exhibited the fastest recovery with transient hyperaccumulation when Pi was replenished. Distinct spatiotemporal responses to Pi availability suggest differences in one or more of the mechanisms that control cytosolic Pi homeostasis. These include uptake from the environment, assimilation to organic forms, metabolic recycling from assimilated forms, sequestration in organelles, and intercellular movement. Although most of these processes are likely to occur simultaneously in a given cell, we were able to distinguish individual contributions of some processes by inhibiting Pi assimilation in the presence and absence of external Pi in combination with short assay times that minimized the effect of its intercellular movement.

When Pi assimilation was blocked in the absence of external Pi (no uptake), we detected rapid increases in cytosolic Pi levels that we attributed to metabolic recycling. Recycling activities consistently reached maximum levels within 10 s, suggesting that additional effects of intercellular movement in this time span would be negligible. However, because reassimilation of Pi liberated from organic forms during this time would also be inhibited, our measures of Pi recycling must be viewed as underestimates. Nevertheless, Pi recycling activity was not uniform in the root. Pi recycling was greater in the basal portion of the root, with equivalent activities in the EZ, DZ, and MR, while less recycling occurred in the apical part of the root, with equivalent activities in the LRC, MZ, and TZ. The basis for this spatial difference in metabolic recycling is unclear, because there was no corresponding effect on cell size or on processes associated with specific developmental zones, e.g. cell division in the MZ and cell elongation in the EZ. It is therefore likely that the two levels of Pi recycling we observed reflect distinct combinations of metabolic activities.

Adding Pi to roots while also inhibiting its assimilation led to increases in cytosolic Pi concentrations beyond those due to metabolic recycling alone. We attributed these additional gains in Pi concentration to uptake, which we detected in cells within the LRC, MZ, and TZ, but not the EZ, DZ, and MR. However, because this measure of Pi uptake is dependent on changes in its concentration, its magnitude is also a function of cytosolic volume. We therefore accounted for differences in cytosolic volumes to evaluate uptake as the amount of newly acquired Pi per cell. On this basis, uptake in the larger cells within the EZ, DZ, and MR may occur at rates equal to or less than those in the smaller, more apical cells. We found that Pi uptake was greatest in the LRC, which is consistent with localization of PHT1-type Pi transporters and the accumulation of total P after exposure of roots to ^33^Pi (Kanno et al., 2016a). Substantial Pi uptake was also detected with nearly equal activity in the MZ and TZ, suggesting that one or more PHT1-type transporters are also active in these zones. Future efforts to extend the live imaging approaches described here to Pi transport mutants may discern cell- or developmental zone-specific contributions of individual transporters.

Although we observed distinct spatial patterns in the root for both uptake and metabolic recycling of Pi, neither of these patterns corresponded with cytosolic Pi concentrations. In contrast, the spatial pattern of vacuolar Pi sequestration suggested that this activity is a major determinant of the cytosolic Pi concentration profile in the root. Under Pi-replete growth conditions, the *pht5;1* mutant, which has a defect in loading Pi into vacuoles (Liu et al., 2016), showed hyperaccumulation of Pi in the cytosol throughout the root except in the TZ. This implies that in wild-type plants, PHT5;1 is developmentally regulated for low activity in the TZ. As a result, cytosolic Pi concentrations are maintained at higher levels in the TZ than in the adjacent developmental zones. The mechanism by which PHT5;1 transport activity is regulated is unknown, but *PHT5;1* transcript abundance does not vary between developmental zones of the root (Brady et al., 2007). Interestingly, overexpression of *PHT5;1* reduced cytosolic Pi levels in the TZ but had no significant effect in the rest of the root. This difference may reflect a thermodynamic limitation for augmenting transport of Pi into vacuoles that already contain high concentrations of Pi.

The TZ is the boundary between dividing (MZ) and elongating (EZ) cells where cellular architecture is reorganized to enable rapid cell elongation (Baluška et al., 1996). It is possible that elevated Pi levels in the TZ are simply a consequence of concomitant vacuolar reorganization. Alternatively, we speculate that TZ-specific vacuolar control of cytosolic Pi levels may influence aspects of Pi signaling to affect cell elongation and cell division in neighboring root regions. Although cells in the TZ have the highest cytosolic Pi concentrations in the root, they also exhibit the greatest concentration change in response to Pi deprivation, ideally positioning these cells to sense fluctuations in Pi availability. Our idea is based on recent findings supporting the idea that the TZ integrates environmental cues, including low-Pi stress, with hormone signals to control cell fate and root growth (Baluška et al., 2010; Di Mambro et al., 2017; Kong et al., 2018). For example, when the root apex encounters low external Pi, cell elongation is rapidly inhibited through apoplastic malate, Fe, and peroxidase-dependent stiffening of the cell wall (Balzergue et al., 2017; Mora-Macías et al., 2017). These processes are regulated through activities of three molecules: the transcription factor SENSITIVE TO PROTON RHIZOTOXICITY (STOP1), which is recruited to the nucleus under low-Pi conditions via an unknown mechanism (Balzergue et al., 2017); LOW PHOSPHATE ROOT1 (LPR1), which mediates ferroxidase activity in the cell wall (Balzergue et al., 2017; Gutierrez-Alanis et al., 2017; Mora-Macías et al., 2017); and CLAVATA3/ENDOSPERM SURROUNDING REGION14 (CLE14) peptide, which is coupled with Fe and callose deposition in the MZ to trigger terminal differentiation and arrest of mitotic activity (Sánchez-Calderón et al., 2005; Balzergue et al., 2017; Gutierrez-Alanis et al., 2017; Mora-Macías et al., 2017). We speculate that low cytosolic Pi levels in the TZ promote nuclear recruitment of STOP1 and thereby modulate subsequent cellular responses to changes in Pi availability. A downstream response to Pi deprivation is the elevated synthesis of the PHR1-regulated VTC4 ascorbate synthase (Mora-Macías et al., 2017). Ascorbate efflux, facilitated by VTC4, could couple with LPR1 ferroxidase activity to complete an Fe redox cycle to produce reactive oxygen species that promote callose deposition in the EZ and MZ (Mora-Macías et al., 2017). In contrast, high Pi concentrations in the TZ would suppress PHR1/SPX1-mediated Pi signaling either directly, through binding SPX1, or indirectly, by affecting the concentration of PP-InsP isoforms, which are high-affinity ligands for SPX1 (Wild et al., 2016; Zhu et al., 2019). Differential control of PHR1/SPX1-mediated Pi signaling in the TZ may tune spatial and/or temporal responses to Pi availability in the EZ and MZ to provide a link between systemic and local Pi signaling pathways. It will therefore be interesting to test the effects and timing of vacuolar Pi sequestration and cytosolic Pi levels in the TZ, as well as vacuolar development in general, on these complex aspects of Pi signaling in roots.

## MATERIALS AND METHODS

### Plant Material and Growth Conditions

Arabidopsis (*Arabidopsis thaliana*) seeds from plants expressing the cpFLIPPi-5.3m Pi sensor (Mukherjee et al., 2015) or a Pi-insensitive control sensor, cpFLIPPi-Null (Banerjee et al., 2016), were grown in 96-well microplates containing 0.5× Murashige and Skoog medium (Murashige and Skoog, 1962) with 0.25% (w/v) Suc and, unless indicated otherwise, 0.25 mm Pi (Pi-replete). All plants carry the *suppressor of gene silencing3-13* (*sgs3-13*) mutation to improve sensor signal intensity and stability (Kumakura et al., 2009; Mukherjee et al., 2015). Plates were incubated in a growth chamber (60% relative humidity, 21°C, and 110 μmol m^−2^ s^−1^ light intensity for a 16-h photoperiod). After 6 d, the seedlings were imaged or transferred to fresh medium as indicated. For longer-term growth, plants were kept on agar-solidified (0.7% [w/v]) Pi-replete medium.

### Live Pi Imaging

Seedlings were mounted on a coverslip in growth medium. A smaller coverslip was placed on top of the root to keep it flat during imaging. Roots were imaged using an inverted Olympus IX81 microscope equipped with a Yokogawa CSU-X1 Spinning Disk confocal unit, an iXon3 897 EMCCD camera (Andor Technology), and a 40× (numerical aperture 1.3) oil immersion objective. Sensor eCFP and FRET-derived cpVenus were detected using 445 nm excitation (70% laser power) and 483/32 nm and 542/27 nm emission, respectively. A 515-nm laser (10% laser power) was used for direct excitation of cpVenus. Cell viability was confirmed using SYTOX orange (Truernit and Haseloff, 2008; Mukherjee et al., 2015). Laser power, electron multiplier gain (5%), preamplifier gain (2.4%), and camera sensitivity were the same for all experiments. Images were analyzed using ImageJ. Background fluorescence (mean fluorescent intensities) for each combination of excitation and emission in untransformed plants was subtracted before further processing. FRET-derived cpVenus emission was corrected for donor spectral bleedthrough and acceptor cross excitation to yield sensitized values as described previously (Banerjee et al., 2016).

### In Vivo Calibration of Pi Sensor FRET Ratios

Cytosolic Pi concentrations in individual cells were manipulated via microinjection (Banerjee, 2017). Borosilicate capillaries (1 mm outer diameter, 0.58 mm internal diameter) containing a filament were pulled then filled with injection buffer (50 mm MOPS-KOH [pH 7.3], 0.5 mm MgCl_2_, and varied concentrations of potassium phosphate buffer [pH 7.3] and potassium gluconate). Potassium gluconate was included as needed to maintain the total potassium ion concentration at 75 mm. Injection buffer also included 1 μm mRuby2 protein (Lam et al., 2012) to demarcate the injected cell and to monitor dispersion of injection buffer. The mRuby2 protein was expressed in bacteria and purified as described (Mukherjee et al., 2015). Filled microinjection needles were fit onto a micromanipulator and connected to an Eppendorf FemtoJet pump. Seedlings were placed on a coverslip and most of the root was covered with wet filter paper (Kunkel, 2015). Epidermal cells in the TZ were impaled, and 20 to 25 pL injection buffer was delivered into the cytosol. The total injection time was 5 s, and cells were imaged within an additional 1.5 s. Six to eight independent cells were injected for each Pi concentration. Injection volume was estimated from mock experiments in which injection buffer was delivered into a puddle of halocarbon oil for the same injection time and then the diameter of the spherical droplets was used to calculate volume. To generate a calibration curve, emission ratio values were plotted versus injected Pi concentrations and data then were fit to a single-site binding isotherm (Gu et al., 2006).

### Pi Recycling, Uptake, and Vacuolar Sequestration

Seedlings were mounted on a coverslip in 0.5× Murashige and Skoog medium without Pi. After initial images were captured, the medium was replaced with 30 μL of medium containing 10 mm NaCN (with or without Pi) and the same cells were imaged. For some experiments, CN and Pi treatments were conducted sequentially.

### Estimation of Cytosolic Volume

Seedlings expressing the cytosolic Pi sensor were mounted in growth medium and Z-stacks were acquired for cpVenus emission using a 40× silicone oil objective (numerical aperture 1.25) and a step size of 0.5 μm to yield a voxel size of 0.06 μm^3^. Background fluorescence was subtracted in a batch. A threshold was set for each slice and regions of interest were drawn to distinguish individual cells. The Voxel Counter ImageJ plugin was used to determine total voxels per cell, which then were converted to picoliters.

### Pi Uptake Assay for Whole Roots

Wild-type and *pht5;1-2* plants expressing cpFLIPPi-5.3m were grown in Pi-replete medium for 6 d then transferred to medium without Pi for 48 h, with replacement of medium every 24 h. Seedlings were then transferred to 0.5 mL of Pi-replete medium in a 12-well plate (12 seedlings per well). Aliquots (15 μL) were withdrawn from each well at the indicated time points in Figure 9B. To measure Pi, each aliquot was mixed with 15 μL assay buffer (50 mm MOPS-KOH [pH 7.3], 50 mm KCl, 0.5 mm MgCL_2_, 1 mg mL^−1^ bovine serum albumin, and 1 μm cpFLIPPi-80u) in a well of a black 384-well plate. After 10 min, fluorescence was measured using a microplate reader (Synergy HT) using excitation at 420/27 nm and emission at 485/20 nm and 540/25 nm. Direct cpVenus excitation was set at 500/20 nm. Emission ratios were converted to Pi concentrations based on an in vitro calibration of cpFLIPPi-80u (Mukherjee et al., 2015). Pi uptake was calculated from the depletion of Pi in the medium and expressed as nanomoles of Pi per seedling.

### Statistical Analyses

ANOVA and Tukey’s honestly significant difference (HSD) mean-separation test were used to evaluate differences in mean emission ratios and Pi concentrations between root developmental zones. The Kolmogorov-Smirnov test (Massey, 1951) was used to assess differences in mean Pi concentrations between CN and CN plus Pi treatments. Student’s *t* test was used to evaluate pairwise comparisons of mean differences in Pi concentrations.

### Accession Numbers

Sequence data from this article can be found in the EMBL/GenBank data libraries under accession numbers At5g23570 (*SGS3*) and At1g63010 (*PHT5;1*). Mutants used in this article can be obtained from the Arabidopsis Biological Resource Center under the following accession numbers: SALK_039005 (*sgs3-13*) and SAIL_96_H01 (*pht5;1-2*).

### Supplemental Data

The following supplemental materials are available.

**Supplemental Figure S1.** Comparisons of fluorescence intensities and emission ratios for cpFLIPPi-5.3m and cpFLIPPi-Null in different root zones.
**Supplemental Table S1.** Effect of altered vacuolar Pi transport on cytosolic and vacuolar Pi concentrations in the root.
